# Impact of information letters on the reporting rate of adverse drug reactions and the quality of the reports: a randomized controlled study

**DOI:** 10.1186/1472-6904-11-14

**Published:** 2011-09-07

**Authors:** Marie-Louise Johansson, Staffan Hägg, Susanna M Wallerstedt

**Affiliations:** 1 Department of Clinical Pharmacology and Regional Pharmacovigilance Centre, Sahlgrenska University Hospital, Gothenburg, Sweden; 2 Department of Drug Research/Clinical Pharmacology, Linköping University, Linköping, Sweden

## Abstract

**Background:**

Spontaneous reporting of adverse drug reactions (ADRs) is an important method for pharmacovigilance, but under-reporting and poor quality of reports are major limitations. The aim of this study was to evaluate if repeated one-page ADR information letters affect (i) the reporting rate of ADRs and (ii) the quality of the ADR reports.

**Methods:**

All 151 primary healthcare units in the Region Västra Götaland, Sweden, were randomly allocated (1:1) to an intervention (n = 77) or a control group (n = 74). The intervention consisted of one-page ADR information letters administered at three occasions during 2008 to all physicians and nurses in the intervention units. The number of ADR reports received from the 151 units was registered, as was the quality of the reports, which was defined as high if the ADR was to be reported according to Swedish regulations, that is, if the ADR was (i) serious, (ii) unexpected, and/or (iii) related to the use of new drugs and not labelled as common in the Summary of Product Characteristics. A questionnaire was administered to evaluate if the ADR information letter had reached the intended recipient.

**Results:**

Before the intervention, no significant differences in reporting rate or number of high quality reports could be detected between the randomization groups. In 2008, 79 reports were sent from 37 intervention units and 52 reports from 30 control units (mean number of reports per unit ± standard deviation: 1.0 ± 2.5 vs. 0.7 ± 1.2, P = 0.34). The number of high quality reports was higher in intervention units than in control units (37 vs. 15 reports, 0.5 ± 0.9 vs. 0.2 ± 0.6, P = 0.048). According to the returned questionnaires (n = 1,292, response rate 57%), more persons in the intervention than in the control group had received (29% vs. 19%, P < 0.0001) and read (31% vs. 26%, P < 0.0001) an ADR information letter.

**Conclusions:**

This study suggests that repeated ADR information letters to physicians and nurses do not increase the ADR reporting rate, but may increase the number of high quality reports.

## Background

Clinical trials contribute greatly to knowledge on drug safety. However, uncommon adverse drug reactions (ADRs) and ADRs in certain patient groups not included in clinical trials, e.g. children and older people with many concomitant diseases and medications, cannot be expected to be detected in these trials. Hence post-marketing surveillance on effects of drugs in clinical practice is essential and spontaneous reporting of ADRs has shown to be an important method to increase drug safety knowledge [1]. In Sweden, physicians, dentists, and nurses are obliged to report (i) serious ADRs, (ii) ADRs not mentioned in the summary of product characteristics (SPC), (iii) ADRs related to the use of new drugs (≤ 2 years on the market) except those labelled as common in the SPC, and (iv) ADRs which incidence seems to increase [2]. Reports concerning the three first points may be most important as far as pharmacovigilance is concerned since they may result in relevant ADR signals, defined as reported information on a possible causal relationship between a drug and an adverse event, the relationship being unknown or incompletely documented previously [3]. Thus, the reporting of ADRs ideally should be focused on such ADRs.

A major limitation of the spontaneous reporting system is that only a small part of all ADRs are reported [4]. A review shows that factors associated with under-reporting include ignorance (only severe ADRs need to be reported), diffidence (fear of appearing ridiculous for reporting merely suspected ADRs), lethargy (e.g. lack of interest or time), indifference (one case from an individual doctor does not contribute to medical knowledge), insecurity (causality between a drug and an adverse event is hard to determine), and complacency (only safe drugs are allowed on the market) [5]. Hence, methods to improve the reporting rate of ADRs could address one or more of these obstacles. Ignorance, diffidence, indifference, insecurity, and complacency can be defeated by education and distribution of drug safety information; methods which have been shown to increase the ADR reporting rate [6-9]. Education may also have positive effects on lethargy, but availability of clinical research assistants may be more effective as far as this obstacle is concerned [10]. Methods which reward individual reporters could also be interesting alternatives to increase the reporting, e.g. lottery tickets [11] or detailed feedback [12]. In addition, allowing other categories of reporters could be beneficial, e.g. nurses [13] and patients/consumers [14].

The methods mentioned above differ in efforts and costs, and there is a need for additional easily managed methods to improve ADR reporting which can be maintained over time without too much efforts or costs. Such a method may be distribution of written information. We have previously shown that such information via e-mail had no apparent effect on the reporting of ADRs, although an increase in the reporting rate in general was noted [8]. From our own experience, we know that e-mails are often overlooked and thus the effect on information via this route, although cheap and easily managed, may have limited effects. In the present study, we hypothesized that written information in the format of a letter administered to health care personnel may have a larger impact on the reporting rate. Indeed, a previous time series analysis has shown positive effects on reporting rate when an ADR bulletin was administered quarterly to physicians [7]. To the best of our knowledge, however, the impact of written ADR information letters administered to healthcare personnel on reporting of ADRs has previously not been evaluated in a randomized controlled study. Moreover, knowledge lacks on the impact of such information on which ADRs are reported, an aspect which is important since some ADRs are more valuable in the pharmacovigilance work, as previously mentioned. The aim of the present study was thus to evaluate if repeated ADR information letters administered to physicians and nurses in primary healthcare units through the secretary of the unit can affect (i) the reporting rate of ADRs and (ii) the quality of the ADR reports.

## Methods

All 151 primary healthcare units in the Region Västra Götaland, Sweden, were randomly allocated (1:1) to an intervention or a control group. A primary healthcare unit generally consists of several general practitioners and nurses who serve patients in a limited geographic area, although patients may choose to attend another unit at their convenience. The units were expected to report ADRs to various extents. Furthermore, in 2007, 63 of the units were included in a randomized controlled trial of repeated e-mails with ADR information [8]. Hence, the allocation was stratified according to number of ADR reports in 2007 and whether or not the unit had received the repeated drug safety e-mails. A person not involved in the study and without knowledge about the study protocol performed the randomization procedure.

The intervention consisted of a one-page ADR information letter which was sent to the secretary of each unit with an instruction letter that it should be distributed to all physicians and nurses at the unit. The number of letters supplied for each unit was estimated based on publicly available information on the staffing of the units, and the secretary was instructed to copy the information letter if more letters were needed.

The ADR information letters were constructed by the authors of this study, who, at the time of the study, all worked at the regional pharmacovigilance centre which serves the Region Västra Götaland. The letters consisted of (i) the heading "ADR Information Letter", (ii) a current case report of an ADR and (iii) instructions on what and how to report [see Additional files 1, 2 and 3]. The letters were sent in January, May, and September 2008.

ADR reports from the included primary healthcare units were extracted from the SWEdish Drug Information System (SWEDIS), the Swedish ADR database where all ADR reports are registered, after being assessed for e.g. causality and seriousness according to the definitions by the World Health Organization [3]. The number of reports from each primary healthcare unit was thus registered, as was the quality of the report, which was defined as high if the ADR was to be reported according to the Swedish regulations on ADR reporting, that is, if the ADR was (i) serious, (ii) unexpected, that is, not labelled in the SPC, and/or (iii) related to the use of new drugs (≤ 2 years on the market) and not labelled as common in the SPC. Trained and experienced staff working at the regional pharmacovigilance centre conducted the assessments.

In January 2009, questionnaires were supplied to the secretaries of the intervention and the control units, to be distributed to all physicians and nurses, using a procedure similar to the one described above. The questionnaire included questions as to whether the ADR information letter had been received and read [see Additional file 4]. The questionnaire was to be answered anonymously, and questionnaires administered to intervention and control units differed in the first letter of one word (capital or lower case), in order to distinguish the origin of the returned questionnaire (intervention or control unit).

### Statistics

Statistical analyses were conducted using SPSS 14.0. Mann-Whitney test was used for between-group comparisons of number of reports per unit. A P-value < 0.05 was considered significant. Where appropriate mean (standard deviation) and median (interquartile range [IQR]) was used.

## Results

A total of 77 primary healthcare units were randomly allocated to the intervention group, and the remaining 74 units to the control group. As for characteristics of the randomized units, the median (IQR) numbers of physicians and nurses working at intervention units were 6 (4-7) and 9 (7-13), respectively. The corresponding numbers for the control units were 5 (4-7) and 9 (6-13), respectively. The reporting rate the year before the intervention was 62 reports from 32 (42%) intervention units, and 55 reports from 31 (42%) control units (mean number of reports per unit ± standard deviation (SD): 0.8 ± 1.4 vs.0.74 ± 1.1, P = 0.93). The number of reports per unit ranged from 0 to 8 (intervention) and 0 to 4 (control). There was no statistically significant difference in the number of high quality reports reported by the intervention and control units (n = 30 vs. n = 19, 0.4 ± 0.8 vs. 0.3 ± 0.6, P = 0.21).

In 2008, a total of 131 reports were received from the participating healthcare units; 79 reports were sent from 37 intervention units and 52 reports from 30 control units (mean number of reports per unit ± SD: 1.0 ± 2.5 vs. 0.7 ± 1.2, P = 0.34, Table 1). The number of reports per unit ranged from 0 to 20 (intervention group) and 0 to 7 (control group).

**Table 1:** Description of the reporting of adverse reactions from the randomized primary healthcare units in 2008

	Control units (n = 74)	Intervention units (n = 77)	P-value
Total number of reports	52	79	

Number of reporting units (% of all units)	30 (40.5%)	37 (48.1%)	

Mean number of reports per unit (± SD)**	0.70 ± 1.21	1.03 ± 2.46	0.34

Total number of high quality reports (% of all reports)*	15 (29%)	37 (48%)	
*Serious*	*4*	*12*	
*Unexpected*	*13*	*20*	
*New drug and not* common ADR*	*4*	*7*	

Mean number of high quality reports per unit (± SD)	0.20 ± 0.57	0.47 ± 0.94	0.048

*A high quality report was defined as a report concerning an ADR which should be reported according to Swedish regulations, that is, an ADR which was (i) serious, (ii) unexpected, and/or (iii) related to the use of new drugs and not labelled as common in the SPC.

**Mean ± SD is presented although the non-parametric Mann Whitney test was used for comparisons between randomization groups, since median (interquartile range) would provide limited information.

ADR, adverse drug reaction; SD, standard deviation; SPC, summary of product characteristics

The number of high quality reports was higher in intervention units than in control units (37 vs. 15 reports, mean number of reports per unit ± SD: 0.5 ± 0.9 vs. 0.2 ± 0.6, P = 0.048, Table 1). Summarized, these reports (n = 52, 40% of all reports) concerned (i) serious ADRs (n = 16 [12% of all reports]), (ii) unexpected ADRs (n = 33 [25% of all reports]), and/or (iii) new drugs (n = 11 [8% of all reports]), as presented in Table 1. The high quality reports concerned 44 substances. Varenicline, acetylsalicylic acid, enalapril, citalopram, and levonorgestrel were reported more than once, and the details of the reports concerning these substances are described in Table 2.

**Table 2:** Description of high quality reports concerning substances reported more than once

Age (years)	Sex	Suspected substance/s	Dose	ADR diagnosis	Serious*	Unexpected*	New drug and not common ADR**	Treatment duration	Time to ADR onset	Positive dechallange	Concomitant medication	Causality assessment*
55	F	Acetylsalicylic acid/caffeine	500/50 mg	Pruritus	N	Y	N	1 dose	Hours	NR	NR	Probable

63	F	Acetylsalicylic acid	NR	Haemorrhagic gastric ulcer	Y	N	N	NR	NR	Y	Candesartan	Possible

80	F	Acetylsalicylic acid	NR	Gastrointestinal haemorrhage	Y	N	N	8 weeks	8 weeks	Died	NR	Possible

56	F	Citalopram	60 mg	Nail disorder	N	Y	N	NR	NR	Medication continued	Folic acid Propiomazine Diazepam Levothyroxine	Possible

78	F	Citalopram Enalapril Dextropropoxyphene Omeprazole Bendroflumethiazide	20 mg 15 mg 150 mg 20 mg 5 mg	Hyponatraemia Confusion	Y	N	N	NR	NR	Medication continued	Dipyridamole Budesonide/ formoterol Tiotropium Cholecalciferol/calcium Macrogol, combinations Vitamin B-complex Paracetamol Cyanocobalamin Sodium picosulfate Ferrous sulphate Levothyroxine Antacids, salt combination Acetylcysteine	Possible

38	F	Enalapril	5 mg	Yawning	N	Y	N	3 weeks	Days	Y	N	Possible

69	F	Enalapril	20 mg	Diplopia	N	Y	N	NR	NR	Medication continued	N	Possible

35	F	Levonorgestrel	NR	Fatigue Myalgia	N	Y Y	N	15 months	Days	Y	Paracetamol Tramadol	Possible

38	F	Levonorgestrel	NR	Ectopic pregnancy	Y	N	N	4 years	4 years	Y	N	Possible

49	M	Varenicline	1 mg	Thrombophlebitis	N	Y	Y	2 months	2 months	NR	Atenolol	Possible

61	F	Varenicline	2 mg	Confusion	N	Y	Y	2 weeks	NR	Y	Dipyridamole Simvastatin	Possible

62	F	Varenicline	NR	Macula-degeneration	N	Y	Y	4 months	5 months	NR	NR	Unclassifiable

66	F	Varenicline	NR	Oedema legs	Y	Y	Y	5 weeks	4 weeks	N	Naproxen Omeprazole Folic acid	Possible

*According to the World Health Organization (WHO)[3]

**Concerning new drugs and not labelled as common in the SPC

ADR, adverse drug reaction; F, female; M, male; N, no; NR, not reported; Y, yes

A total of 845 physicians and 1,423 nurses worked in the primary healthcare units. A total of 1,292 questionnaires were duly filled and returned. The response rate was therefore 57% (physicians, n = 556; nurses, n = 711; other professions, n = 17 [these were not intended to receive and respond to the questionnaire, but did so anyway]). A total of 300 respondents reported having received at least one ADR information letter during 2008 (23%), and 362 (28%) had read at least one ADR information letter during the year. More persons in the intervention group than in the control group had received (29% vs. 19%, P < 0.0001) and read (31% vs. 26%, P < 0.0001) an ADR information letter during 2008. In the intervention group, more physicians than nurses had received (36% vs. 28%, P < 0.015) but not read (36% vs. 37%, P = 0.89) the ADR information letter.

## Discussion

In the present study, no effect of repeated one-page ADR information letters on the overall reporting rate could be detected. The results are in contrast to the findings by Castel *et al.*, who reported an increased reporting rate after distribution of a drug safety bulletin [7]. One explanation for the divergent findings may be that the methodologies to evaluate differences in reporting rate differ between the studies; we used a randomized controlled design whereas Castel *et al. *used a time series methodology. Nevertheless, other interventions may be more useful when the number of ADR reports is to be increased, such as education [6,9] or detailed feedback to the reporting physician [12].

Our results show that more high quality reports were received from intervention than control units, that is, the reports more often concerned ADRs which should be reported according to Swedish regulations. Thus, repeated ADR information letters may represent a valuable means to increase the number of reports which should actually be reported. One may speculate that a combination of such letters with an educational intervention may be even more effective as regards this aspect, since the latter also has been shown beneficial [6]. Indeed, from a pharmacovigilance perspective, it is of importance that ADR reporting is focused on ADRs which are most likely to contribute to increased drug safety knowledge. ADR reports concerning well-known non-serious conditions may thus be of limited value since these contribute little to ADR signals. On the contrary, these reports constitute background noise, which may make detection of ADR signals more difficult at least as far as statistical signal detection methods within ADR databases are concerned, e.g. Proportional Reporting Ratios (PRR) [15] and Bayesian Confidence Propagation Neural Network (BCPNN) [16].

Varenicline was the most frequently reported substance in high quality reports. This substance was registered for smoking cessation in 2006 and thus the reports concerned a new drug. In addition, these reports also concerned unexpected ADRs. Indeed, the majority of high quality reports concerned unexpected ADRs. This finding may not be surprising since primary healthcare personnel probably observe serious ADRs less frequently than hospital personnel due to the definition of a serious ADR; any untoward medical occurrence that, at any dose (i) results in death; (ii) requires inpatient hospitalization or prolongation of existing hospitalization; (iii) results in persistent or significant disability/incapacity; or (iv) is life-threatening [3].

Interestingly, 84 out of 151 primary healthcare units (56%) did not report any ADR during 2008. This finding supports a high degree of under-reporting [4], and may indicate that primary healthcare personnel is an important target for interventions for improved reporting of ADRs.

Significantly more questionnaire responders had received and read the ADR information letters in the intervention group. However, the figures were generally low, indicating that ADR information letters are not prioritized reading for healthcare personnel in clinical practice. Interestingly, many questionnaire responders in the control units reported having received and read the ADR information letter. The intervention thus seems to have spilled over to the control units. Physicians and nurses may work in more than one primary healthcare unit, that is, both in the intervention group and in the control group. Furthermore, the units all belong to the same organization and information may thus easily pass from one unit to another. Another explanation for the finding that the ADR information letters were read by personnel in the control units is that ADR information from other sources, e.g. the pharmaceutical industry, may have been administered to the primary healthcare during 2008.

An important limitation of the present study is the small number of reports received from the small number of primary healthcare units. Indeed, given the available number of primary healthcare units in the region and the final results, the power of the present study to detect differences between the groups ended at 17%.

Another limitation is that our definition of quality of ADR reports was quite strict and related only to the Swedish regulations on ADR reporting. Thus, the information content of the report as regards other important aspects were not evaluated, such as factors of importance for the assessment of the strength of the relationship between the drug/s and the event/s, i.e. time to ADR onset, and response to dechallenge and rechallenge.

## Conclusions

Repeated ADR information letters to physicians and nurses was not found to increase the ADR reporting rate, However, such an intervention may still be favorable from a pharmacovigilance perspective since it resulted in an increased number of reports concerning ADRs which should be reported according to Swedish regulations.

## Competing interests

The authors declare that they have no competing interests.

## Authors' contributions

MLJ carried out the acquisition of data. SMW conceived the study, performed the statistical analyses and drafted the manuscript. All authors contributed to the design of the study, revised the manuscript, and read and approved the final manuscript.

## Pre-publication history

The pre-publication history for this paper can be accessed here:

http://www.biomedcentral.com/1472-6904/11/14/prepub

## Supplementary Material

Additional file 1 **ADR information letter I**. The first ADR information letter sent to physicians and nurses in the intervention units (translated to English). Click here for file

Additional file 2 **ADR information letter II**. The second ADR information letter sent to physicians and nurses in the intervention units (translated to English). Click here for file

Additional file 3 **ADR information letter III**. The third ADR information letter sent to physicians and nurses in the intervention units (translated to English). Click here for file

Additional file 4 **Questionnaire**. Questionnaire sent to physicians and nurses in intervention and control units (translated to English). Click here for file
