# Supplementary material for: Impact of information letters on the reporting rate of adverse drug reactions and the quality of the reports: a randomized controlled study
Source: BMC Clin Pharmacol. 2011 Sep 7;11:14. doi: 10.1186/1472-6904-11-14 (PMC3182972; doi:10.1186/1472-6904-11-14)
Supplement: Additional file 1 — ADR information letter I. The first ADR information letter sent to physicians and nurses in the intervention units (translated to English). [file 1472-6904-11-14-S1.DOC]

**ADR Information Letter**

**15 February 2008**

Doctors and nurses who meet patients in clinical practice can observe adverse drug reactions. When you report such reactions, others may benefit from your experience, and drug safety knowledge is increased. With this newsletter from the Regional Pharmacovigilance Centre in Western Sweden, doctors and nurses will receive alerts on serious and not generally known adverse drug reactions reported in the region.

Marie-Louise Johansson Staffan Hägg Susanna Wallerstedt

Reg. nurse Consultant/ ass prof Specialist physician /PhD

**Two recent case reports from the Regional Pharmacovigilance Centre in Western Sweden**

In several cases, psychiatric symptoms during use of Champix (varenicline) have been reported to regional pharmacovigilance centres in Sweden. In two recent cases, depression and suicidality, respectively, were reported. In these reports, women developed depressive symptoms after a relatively short-term treatment. In one case, the depressive symptoms occurred concomitantly with a drug intoxication, and in the second case, they occurred concomitantly with suicidal thoughts. Psychiatric disorders such as abnormal dreams, insomnia, panic attacks, bradyphrenia, abnormal thinking, and mood swings are listed in FASS. In December 2007 the European Medicines Agency scientific committee (CHMP) issued a strengthened warning for the drug due to psychiatric adverse drug reactions such as suicide ideation and suicide attempts.

**Each adverse drug reaction report is important!**

**Report the following:**

1. All serious adverse drug reactions

2. All adverse drug reactions that are not in FASS

3. All adverse drug reactions that seem to increase in frequency

4. For new drugs (see last page of the “blue letter" from the Medical Product Agency) - all adverse drug reactions that are not listed as common in FASS

**It is easy to report:**

Send a copy of the case records to the Regional Pharmacovigilance Centre in Western Sweden, Sahlgrenska University Hospital, 413 45 Gothenburg

or

Complete the adverse drug reaction form found at the back of FASS or at the website of Region Västra Götaland
