# Supplementary material for: Impact of information letters on the reporting rate of adverse drug reactions and the quality of the reports: a randomized controlled study
Source: BMC Clin Pharmacol. 2011 Sep 7;11:14. doi: 10.1186/1472-6904-11-14 (PMC3182972; doi:10.1186/1472-6904-11-14)
Supplement: Additional file 2 — ADR information letter II. The second ADR information letter sent to physicians and nurses in the intervention units (translated to English). [file 1472-6904-11-14-S2.DOC]

**ADR Information Letter**

**5 May 2008**

Doctors and nurses who meet patients in clinical practice can observe adverse drug reactions. When you report such reactions, others may benefit from your experience, and drug safety knowledge is increased. With this newsletter from the Regional Pharmacovigilance Centre in Western Sweden, doctors and nurses will receive alerts on serious and not generally known adverse drug reactions reported in the region.

Marie-Louise Johansson Staffan Hägg Susanna Wallerstedt

Reg. nurse Consultant/ ass prof Specialist physician /PhD

**A recent case report from the Regional Pharmacovigilance Center in Western Sweden**

In a case recently reported to the Regional Pharmacovigilance Center, Western Region, a middle-aged woman treated with aloe vera for several months is described. She stated that she probably had overdosed aloe vera lately. Elevated liver enzymes were found at the primary healthcare centre (AST 14 μkat/L, ALT 30 μkat/L, ALP and bilirubin were normal). No obvious reasons for these findings were identified. When the woman stopped the treatment with aloe vera the liver enzyme values normalized.

In the Swedish pharmacovigilance database there is another case of hepatitis associated with aloe vera treatment. In the WHO international pharmacovigilance database, there are 3 additional cases of transaminase elevation and 2 cases of hepatitis.

Do not forget to ask your patient about intake of herbal medicines when asking about medications!

**Each adverse drug reaction report is important!**

**Report the following:**

1. All serious adverse drug reactions

2. All adverse drug reactions that are not in FASS

3. All adverse drug reactions that seem to increase in frequency

4. For new drugs (see last page of the “blue letter" from the Medical Product Agency) - all adverse drug reactions that are not listed as common in FASS

**It is easy to report:**

Send a copy of the case records to the Regional Pharmacovigilance Centre in Western Sweden, Sahlgrenska University Hospital, 413 45 Gothenburg

or

Complete the adverse drug reaction form found at the back of FASS or at the website of Region Västra Götaland
