# Supplementary material for: Impact of information letters on the reporting rate of adverse drug reactions and the quality of the reports: a randomized controlled study
Source: BMC Clin Pharmacol. 2011 Sep 7;11:14. doi: 10.1186/1472-6904-11-14 (PMC3182972; doi:10.1186/1472-6904-11-14)
Supplement: Additional file 4 — Questionnaire. Questionnaire sent to physicians and nurses in intervention and control units (translated to English). [file 1472-6904-11-14-S4.DOC]

Questionnaire

**I am a/an**

□ licensed physician □ nurse with prescribing authority

□ unlicensed physician □ nurse without prescribing authority

□ other………………………………..

**I am a** □ woman □ man

**I have received** □ one □ two □ three □ no **newsletter/s from the Regional Pharmacovigilance Centre in Western Sweden in 2008.**

**I have read** □ one □ two □ three □ no **newsletter/s from the Regional Pharmacovigilance Centre in Western Sweden in 2008.**

***Thank you for your participation!***
